# Annual acknowledgement of manuscript reviewers

**DOI:** 10.1186/1475-2859-13-18

**Published:** 2014-02-06

**Authors:** Antonio Villaverde

**Affiliations:** 1 Institut de Biotecnologia i de Biomedicina, Universitat Autònoma de Barcelona Bellaterra, Barcelona 08193, Spain; 2 Department de Genètica i de Microbiologia, Universitat Autònoma de Barcelona, Bellaterra, Barcelona 08193, Spain; 3 CIBER de Bioingeniería, Biomateriales y Nanomedicina (CIBER-BBN), Bellaterra, Barcelona 08193, Spain

## Abstract

**Contributing reviewers:**

The Editors of Microbial Cell Factories would like to thank all our Reviewers who have contributed to the journal in Volume 12 (2013).

## 

Claus Aagaard

Denmark

Randa A Abusham

Malaysia

Pratul Agarwal

United States of America

George Aggelis

Greece

Ibrar Ahmed

New Zealand

Pietro Alifano

Italy

Ana Paula Alonso

United States of America

Hal Alper

United States of America

Diletta Ami

Italy

Ricardo Amils

Spain

Evelina Angov

United States of America

Jozef Anne

Belgium

Anjali Apte-Deshpande

India

Shota Atsumi

United States of America

Bernhard Auer

Austria

Francesc X Aviles

Spain

Vasco Azevedo

Brazil

Loredana Baccigalupi

Italy

Fernanda Badotti

Brazil

Mohammed Bahey-El-Din

Egypt

Antonio O Ballesteros

Spain

Richard Baltz

United States of America

François Baneyx

United States of America

Ashok Bankar

India

Xiaoming Bao

China

Paolo Barghini

Italy

Rodolphe Barrangou

United States of America

Enrico Baruffini

Italy

Altaf Basta

Egypt

Karl Bayer

Austria

Michele Bellucci

Italy

William Bentley

United States of America

Ales Berlec

Slovenia

Luis G Bermudez-Humaran

France

Maurizio Bettiga

Sweden

Jean-Michel Betton

France

Sanjoy K Bhattacharya

United States of America

Frédérique Bidard

France

Rebekka Biedendieck

Germany

Roslyn Bill

United Kingdom

Philippe Billiald

France

Bernward Bisping

Germany

Jens Bitzer

Germany

Lars Mathias Blank

Germany

Bastian Blombach

Germany

Léonie Boender-van-Dijk

Netherlands

Eckhard Boles

Germany

Francisco Bolivar

Mexico

Monique Bolotin-Fukuhara

France

Irina Borodina

Denmark

Alejandra Bosch

Argentina

Frederic Bourgaud

France

Daniel Bracewell

United Kingdom

Luca Brambilla

Italy

Paola Branduardi

Italy

Trygve Brautaset

Norway

Michael Breitenbach

Austria

Ryszard Brzezinski

Canada

Markus Buchhaupt

Germany

Jochen Büchs

Germany

Tim Bull

United Kingdom

Jeremy Burton

Canada

Pietro Buzzini

Italy

Bernadette Byrne

United Kingdom

Zeynep Petek Cakar

Turkey

Pinar Calik

Turkey

Susana Campoy

Spain

Larissa Canilha

Brazil

Elisabetta Carata

Italy

Vicelma Cardoso

Brazil

Lisa Carius

Germany

Francisco Carrau

Uruguay

Eduardo A Ceccarelli

Argentina

Eda Çelik-Akdur

Turkey

Hyung Joon Cha

Korea, South

Keith Chater

United Kingdom

Francisco P Chávez

Chile

Guo-Qiang Chen

China

Rachel Chen

United States of America

Wilfred Chen

United States of America

Yuh Min Chook

United States of America

Victor Cifuentes

Chile

Donatella Cimini

Italy

Patrick Cirino

United States of America

Thomas Clarke

United Kingdom

Jose Luis Corchero

Spain

Aldo Corsetti

Italy

Naima Gisela Cortes-Perez

France

Don Cowan

South Africa

Russell Cox

Germany

Roger Cripps

United Kingdom

Roy Curtiss

United States of America

Jean-Marc Daran

Netherlands

Carla de Carvalho

Portugal

Jan-Willem de Gier

Sweden

Victor de Lorenzo

Spain

Ario de Marco

Slovenia

Kristof De Schutter

Belgium

Gloria del Solar

Spain

Marizela Delic

Austria

Matthew DeLisa

United States of America

Frank Delvigne

Belgium

Josef Deutscher

France

Petr Dmitriev

France

Silvia Doglia

Italy

Mike Domach

United States of America

Pedro Domingos

Portugal

Martin Dragosits

Austria

David Drew

United Kingdom

Paul Dyson

United Kingdom

Suhelen Egan

Australia

Lothar Eggeling

Germany

Hani El-Nezami

Hong Kong

Sven-Olof Enfors

Sweden

Adelfo Escalante

Mexico

Gaetan Faubert

Canada

Fabio Fava

Italy

Victor Fedorenko

Ukraine

Ana Ferreira

Portugal

Pau Ferrer

Spain

Stephen Fong

United States of America

Luis Fonseca

Portugal

Annie Frelet-Barrand

France

Lars Frelin

Sweden

Karl Friehs

Germany

Niels-Ulrik Frigaard

Denmark

Chikara Furusawa

Japan

Chikara Furusawa

Japan

Matthew Gage

United States of America

Jose Galgani

Chile

Giuseppe Gallo

Italy

Ravi Ganapathy

India

Jose Luis Garcia

Spain

Elena Garcia-Fruitos

Spain

Teresa Garcia-Martinez

Spain

Brigitte Gasser

Austria

George Georgiou

United States of America

Johannes Gescher

Germany

Nasser Ghaemi

Iran

Emilia Ghelardi

Italy

Isidre Gibert

Spain

Opher Gileadi

United Kingdom

Monika Glinkowska

Poland

Yong Jun Goh

United States of America

Digambar Gokhale

India

Katsuya Gomi

Japan

Ramon Gonzalez

United States of America

Marie-F Gorwa-Grauslund

Sweden

Guillermo Gosset

Mexico

Astrid Groot

Netherlands

Víctor Gabriel Guadalupe Medina

Netherlands

Maria Gullo

Italy

Paramasamy Gunasekaran

India

Yingying Guo

China

Hiroshi Habe

Japan

Wataru Hakamata

Japan

Mari Häkkinen

Finland

Mee-Jung Han

Korea, South

Colin Harwood

United Kingdom

Rajni Hatti Kaul

Sweden

Michael Hecker

Germany

Elmar Heinzle

Germany

Senta Heiss-Blanquet

France

Klaas Jan Hellingwerf

Netherlands

Martín Hernández

Argentina

Enrique Herrero

Spain

Wolfgang Rene Hess

Germany

Anthony Hickey

United States of America

Lihua Hou

China

Gerardo Huerta-Beristain

Mexico

William J Hunter

United States of America

Tuomas Huovinen

Finland

Michael Hust

Germany

Zoya Ignatova

Germany

Mehmet Inan

Turkey

Rachele Isticato

Italy

Sylwia Jafra

Poland

Yu-Sin Jang

Korea, South

Zhengbing Jiang

China

Yong-Su Jin

United States of America

Song Jinzhu

China

Toru Jojima

Japan

Paula Jouhten

Finland

Matthieu Jules

France

Naheed Kaderbhai

United Kingdom

Héla Kallel

Tunisia

Hyun Ah Kang

Korea, South

Joan Kelly

Australia

Byoungjin Kim

Korea, South

Tominori Kimura

Japan

Maria Klapa

Greece

Mario Klimacek

Austria

Dennis Kobelt

Germany

Michael Koepke

New Zealand

Kari Koivuranta

Finland

Jan Kok

Netherlands

Akihiko Kondo

Japan

Maria Kosseva

China

Athanasios Koutinas

Greece

Thijs Kouwen

Netherlands

Akos T Kovacs

Germany

Regina Kratzer

Austria

Anastasia Krivoruchko

Sweden

Christian P Kubicek

Austria

Thomas Kuhlman

United States of America

Maria Laaberki

France

Alvaro R Lara

Mexico

Gen Larsson

Sweden

Pyung Cheon Lee

Korea, South

Anne L'Hernault

United Kingdom

Wei Lian

United States of America

Wei Liao

United States of America

Zhanglin Lin

China

Robert Linhardt

United States of America

Mirjana Liovic

Slovenia

Shuang-Jiang Liu

China

Jaime Lizardi-Mendoza

Mexico

Cecilio Lopez-Galindez

Spain

Christian Löser

Germany

Ming-Yeou Lung

Taiwan

Andriy Luzhetskyy

Germany

Gary Lye

United Kingdom

Daniel MacEachran

United States of America

Robert Mach

Austria

Valeria Mapelli

Denmark

Marc J.C. Fischer

France

Gregory Marczynski

Canada

Gergely Maroti

Hungary

Juan F Martin

Spain

Alfredo Martinez

Mexico

Sergey Mashko

Russian Federation

Lluis Masip

Spain

Luke Masson

Canada

Diethard Mattanovich

Austria

Joseph McCormick

United States of America

Brian McNeil

United Kingdom

Adam Meadows

United States of America

Jochen Meens

Germany

Carmen Méndez

Spain

Ivan Mijakovic

Sweden

Natalia Minaeva

Russian Federation

Bushra Mirza

Pakistan

Shusaku Mizukami

Japan

Axel Mogk

Germany

Diego Mora

Italy

Leonora Moreira

Brazil

Krishna Jyoti Mukherjee

India

Susann Müller

Germany

Rosario Muñoz

Spain

Kenneth Narva

United States of America

Juana Maria Navarro Llorens

Spain

Darren Nesbeth

United Kingdom

Peter Neubauer

Germany

Helena Nevalainen

Australia

Elke Nevoigt

Germany

Jens Nielsen

Denmark

Pablo Ivan Nikel

Spain

Stephan Noack

Germany

Philippe Noirot

France

Kevin O Connor

Ireland

Michal Obuchowski

Poland

Shigeru Okada

Japan

Carlos Olano

Spain

Sahlan Ozturk

Turkey

Laura Palomares

Mexico

Gianni Panagiotou

Hong Kong

Sven Panke

Switzerland

Deepak Pant

Belgium

Maria Papagianni

Greece

Leandro Papinutti

Argentina

Eugenio Parente

Italy

Luís Passarinha

Portugal

Flavia Passos

Brazil

Miroslav Patek

Czech Republic

Christie Peebles

United States of America

Roberto Pérez-Torrado

Spain

Clemens Peterbauer

Austria

Spela Peternel

Slovenia

Brian Pfleger

United States of America

Jean-Christophe Piard

France

Karen Polizzi

United Kingdom

Isabelle Poquet

France

Danilo Porro

Italy

Anna Maria Puglia

Italy

Peter J Punt

Netherlands

Qingsheng Qi

China

Jian Qin

Bahrain

Octavio Ramirez

Mexico

Ursula Rinas

Germany

Frank Robb

United States of America

Gilles Robitaille

Canada

Changhyun Roh

Korea, South

Susana Romero-Garcia

Mexico

Robyn Roth

South Africa

Maurizio Ruzzi

Italy

Margherita Sacco

Italy

Yumiko Sakuragi

Denmark

Jose Salas

Spain

Markku Saloheimo

Finland

Ramón I Santamaría

Spain

Muthuswamy Sathishkumar

Singapore

Michael Sauer

Austria

David Savage

United States of America

Erkki Savilahti

Finland

Thomas Scanlon

United States of America

Wilhelm Schaefer

Germany

Peter Scharff-Poulsen

Denmark

Herb Schellhorn

Canada

Chiara Schiraldi

Italy

Wolfgang Schumann

Germany

Zorita Sconta

Romania

Nunzia Scotti

Italy

Maria Mercedes Segura

Spain

Bernhard Seiboth

Austria

Ryan Senger

United States of America

Enrique Seoane-Vazquez

United States of America

Diana Isabella Serrazanetti

Italy

Shaobin Shang

United States of America

Joseph Shiloach

United States of America

Verena Siewers

Sweden

Jean-Claude Sirard

France

Thomas Smithgall

United States of America

Bernhard Sonnleitner

Switzerland

Giuseppe Spano

Italy

Edzard Spillner

Germany

Georg Sprenger

Germany

Ganesh Sriram

United States of America

Boris Stambuk

Brazil

Matthias Steiger

Austria

Piotr Stepnowski

Poland

Gerald Striedner

Austria

Masashi Suzuki

Japan

Sutipa Tanapongpipat

Thailand

Yinjie J Tang

United States of America

Chandresh Thakker

India

Tran Thanh

Malaysia

Kate Thodey

United States of America

Oleg Tolmachov

United Kingdom

Monica Trif

Germany

Shen-Long Tsai

Taiwan

Hiroshi Tsujibo

Japan

Cagla Tukel

United States of America

Sedef Tunca Gedik

Turkey

Charles Turnbough

United States of America

Maria Luisa Tutino

Italy

Keith Tyo

United States of America

Stéphane Uroz

France

Francisco Valero

Spain

Paul MP Van Bergen en Henegouwen

Netherlands

Johan Van Hylckama Vlieg

France

Erick Vandamme

Belgium

Mario Varcamonti

Italy

Jean-Pierre Vartanian

France

Salvador Ventura

Spain

Walter Vetter

Germany

Koyadan Kizhakkedath Vijayan

India

Nicola Volpi

Italy

Tobias von der Haar

United Kingdom

Gerard Wall

Ireland

Xiu-Ling Wang

China

Mark Welch

United States of America

Randall Weselake

Canada

Ruud Weusthuis

Netherlands

Marilyn Wiebe

Finland

Nick Wierckx

Germany

Annegret Wilde

Germany

Catherine Winder

United Kingdom

Christoph Wittmann

Germany

Xin-Hui Xing

China

Hideki Yamaji

Japan

Hiroya Yurimoto

Japan

Rintze Zelle

United States of America

Huimin Zhao

United States of America

Manfred Zinn

Switzerland

